# #Blessed: the moderating effect of dispositional gratitude on the relationship between social comparison and envy on Instagram

**DOI:** 10.3389/fpsyg.2023.1159999

**Published:** 2023-10-20

**Authors:** Stella Kaminger, Leopold Helmut Otto Roth, Anton-Rupert Laireiter

**Affiliations:** ^1^Faculty of Psychology, Institute for Clinical and Health Psychology, University of Vienna, Vienna, Austria; ^2^Faculty of Psychology, Institute of Department of Occupational, Economic, and Social Psychology, Motivation Psychology, University of Vienna, Vienna, Austria; ^3^Division of Psychotherapy and Gerontopsychology, Department of Psychology, University of Salzburg, Salzburg, Austria

**Keywords:** dispositional gratitude, social comparison, benign envy, malicious envy, social media, Instagram

## Abstract

**Introduction:**

The role of dispositional gratitude as a positive psychological resource and prosocial personality trait in real life interactions militates in favor of its introduction to the research field of social media.

**Methods:**

Based on a literature review of the previously studied relationship of dispositional gratitude with social comparison and envy in offline settings, a twofold moderation model was proposed and quantitatively tested in a cross-sectional sample of *N* = 268 Instagram users aged between 18 and 40 years. Additionally, the dual conceptualization of benign and malicious envy was scrutinized by validating its respective connections with affective outcomes and inspiration on Instagram.

**Results and discussion:**

Dispositional gratitude serves as a protective factor when using Instagram by significantly mitigating the relationship of social comparison and malicious as well as general envy on Instagram. Furthermore, the results support the more nuanced understanding of envy as a dual construct in the face of social media use.

## 1. Introduction

Blog posts with titles like “#Blessed: Why it’s not gratitude if you need to advertise it on social media” (Theresa Christine, 2014), “How to practice gratitude without feeling like an Instagram cliché” (Hoff, 2019), and “How gratitude fixed my relationship with Instagram” (Coyne, 2018) indicate the relevance of positive-psychological research for the area of social media. As noted in previous work, social media use can exhibit both positive and negative characteristics with potentially favorable as well as harmful effects on the user’s well-being (Fox and Moreland, 2015; Radovic et al., 2017; Verduyn et al., 2017). Therefore, it is essential to explore the circumstances and mechanisms of both outcomes to promote future beneficial usage behavior. Especially, regarding the increasing number of social media users and the related psychological costs, variables from Positive Psychology show the potential to function as protective factors when using social media (Pantic, 2014; Hansen, 2015; Winata and Andangsari, 2017). For this purpose, the current paper proposes the implementation of gratitude into the research field of social media by exploring its association with social comparison and envy.

### 1.1. Gratitude and social media

Whereas state gratitude refers to the emotional response to a blessing received, trait gratitude considers a habitual and constant characteristic and tendency to experience gratitude with higher frequency and intensity (Wood et al., 2008). Significant to a broader conception of dispositional gratitude is the propensity to appreciate consciously the positive aspects of life and to consider one’s own abilities and possessions as valuable gifts (McCullough et al., 2002; Wood et al., 2010; Winata and Andangsari, 2017). Regarding the aim of integrating gratitude into the research context of social media, the social component of gratitude is especially relevant. To experience as well as to express gratitude strengthens interpersonal relationships and functions as a social engine and amplifier of prosocial behavior (McCullough et al., 2008; Blickhan, 2018). Inherently, gratitude is considered an intrinsic value and the counterpart of extrinsic demands such as materialism, fame, and possession (Froh et al., 2011). There is a growing body of research on the moderating effect of dispositional gratitude in the context of social variables. In this light, dispositional gratitude was found to downsize negative health consequences associated with a lower socioeconomic status (Hartanto et al., 2018). Dispositional gratitude further serves as a moderator when looking at affective needs and victimization frequency in romantic interpersonal relationships (Griffin et al., 2016). From a meta-analytic point of view, dispositional gratitude is described as an especially meaningful trait in predicting well-being (Portocarrero et al., 2020). Despite the positive effects of gratitude in a variety of domains, its implementation in the research context of social media so far is rather sparse. The existing evidence indicates that the potential of dispositional gratitude as a resource in offline-contexts can be transferred to online-interactions. The reciprocity of gratitude, for instance, applies to an online-context too and is present toward contacts that already existed offline (Godawa et al., 2019). Social media users with higher dispositional gratitude undergo less social comparison and exhibit an overall lower tendency to compare with others socially (Winata and Andangsari, 2017). Moreover, a gratitude intervention on Instagram was found to enhance the users’ dispositional gratitude. Due to its interactive features and the social mechanisms behind sharing pictures, Instagram is considered a platform potentially reinforcing gratitude (Koay et al., 2020). On the downside, the platform focuses on aesthetic enhancement by encouraging the use of filters, which in turn favors a strong positivity norm and impels users to mainly share positive and optimized aspects of their lives. As a result, Instagram promotes passive usage habits and almost continuous social comparison processes (Lup et al., 2015). By fostering social comparison, social media use further relates to feelings of envy and decreased well-being (Krasnova et al., 2013; Lange and Crusius, 2015; Verduyn et al., 2015).

### 1.2. The concept of envy

Generally, envy refers to the painful experience of the perceived superiority of others and is considered a frequent cause of dissatisfaction and decreased well-being, as it results from aligning one’s self-worth with the comparison to others (Smith, 2007). Theoretically, two conceptualizations of envy can be differentiated: in the *unitary approach*, envy genuinely entails feelings of pain and hostility and yet possibly triggers positive as well as negative reactions. To do justice to this twofold characteristic of envy, the *dual approach* distinguishes two different forms of envy. Accordingly, *benign envy* aims at reducing the superiority of the envied person by motivating them to catch up, whereas *malicious envy* does so by downgrading, disparaging, and harming the envied (Crusius et al., 2020). Therefore, benign envy is accompanied by active emulation and malicious envy comes along with aggression (Lange et al., 2018). Empirical research as well as factor analyses reveal two inherent factors with weak intercorrelations (*r_*s*_* = −0.07 to 0.32; Lange and Crusius, 2015), hinting at the existence of two forms of envy (Crusius et al., 2020).

To come to terms with this recent debate, we define envy here in a general form as well as in its dual specification for a more differentiated perspective. As previously noted, and generally implemented in social media research, the consideration of the envy subtypes permits an in-depth analysis and reflects the different social media usage outcomes as potentially harmful or beneficial for the users (van de Ven, 2016; Wu and Srite, 2021). Benign envy induces positive outcomes such as inspiration (Meier and Schäfer, 2018), elevated goal setting, better performance (Lange and Crusius, 2015) and positive affect (van de Ven et al., 2009; Braun et al., 2018; Meier and Schäfer, 2018). Malicious envy is associated with “schadenfreude” (Lange et al., 2018), counterproductive work methods (Braun et al., 2018), hostility (Crusius et al., 2020), and negative emotions (Braun et al., 2018). These connections are not applicable to the other form of envy, respectively (Crusius et al., 2020).

When transferring the dual understanding of envy to the research area of gratitude, the findings emphasize the complexity of envy as a psychological phenomenon: Gratitude is defined as a counterpart of and an incompatible characteristic to envy (McCullough et al., 2002). The dual conception of envy allows a more detailed view, with gratitude being a positive predictor of benign and a negative predictor of malicious envy (Xiang et al., 2018).

### 1.3. The current study

Considering that social media use contributes on the one hand positively and on the other hand negatively to users’ well-being, it is especially relevant to expand our knowledge on traits and behaviors inhibiting negative usage habits and promoting positive outcomes (Hansen, 2015). Dispositional gratitude might help to gain new insights into individual usage styles. The relationship between gratitude and social comparison as well as envy suggests that dispositional gratitude might be decisive in whether social media use leads to positive rather than negative consequences. To test this assumption empirically, a twofold moderation model is proposed.

Current research shows that both types of envy are closely associated with social comparison processes (Meier and Schäfer, 2018). Dispositional gratitude is related negatively to the social comparison orientation of social media users (Winata and Andangsari, 2017) and in addition, serves as a positive predictor of benign and as a negative one of malicious envy (Xiang et al., 2018). Taken together, these findings strongly support the assumptions of our moderator model. We therefore assume that dispositional gratitude positively moderates the relationship of social comparison and benign envy and negatively that of social comparison and malicious envy. As a complementary *post hoc* analysis, we explore the moderating effect of dispositional gratitude on the relationship of social comparison and general envy on Instagram. In the context of our model, social comparison processes can operate in two ways, as a general and a specific phenomenon. The first one targets a common trait and the second one relates to Instagram use in specific. For validating the dual conception of envy, we look at their relationships with inspiration on Instagram and positive and negative emotions, supposing that benign envy positively predicts inspiration and positive affect and malicious envy shows no relationship with inspiration and a positive one with negative affect.

## 2. Materials and methods

### 2.1. Sample

The required sample size for detecting a small to medium effect (*f*^2^ = 0.1) with a given power of 0.8 was computed using G*Power 3.1 (Faul et al., 2009), which recommended a minimum number of 114 individuals. In view of range restriction in moderation analyses and in orientation toward existing studies, we collected *ad hoc* data of 303 Instagram users, which resulted in a sample size of 268 Instagram users after the exclusion of incomplete datasets and four cases which completed the survey in less than 4 min. The observed power for each model was computed and can be found on OSF.^1^ Following the inclusion criteria of young and emerging adulthood (18–40 years; Berk, 2011), our sample shows a mean age of 22.84 years (SD = 3.85, min. = 18, max. = 39). Addressing participants in this younger age segment ensured a representation of Instagram users that are especially concerned with self-branding and impression-management, which in turn makes social comparison processes more prevalent (Alfasi, 2019). Further recruitment criteria included people at least occasionally using Instagram and exhibiting sufficient German language skills for questionnaire handling. The majority of the resulting sample identified as female (63.05%) followed by male (36.19%) and two participants identifying as diverse (non-binary; 0.74%). We did not distinguish between gender and sex and did not measure the alignment between the two in this study. Future studies, aiming to target sex and/or gender differences regarding our hypothesis should include the respective measures to allow respective comparisons. Additional descriptive data on the sample is presented in Table 1.

**TABLE 1:** Descriptive details on sample (*N* = 268).

Country	
Austria	73.13%
Germany	20.14%
Other	6.73%
**Relationship**	
Single	50.00%
Relationship	50.00%
**Education**	
No high school	5.22%
High school	69.40%
University	25.38%
Followers (median)	300
Following (median)	250

### 2.2. Measures

To test our hypotheses, we collected data from each participant with six psychometric scales. Gratitude was measured using the *Gratitude Questionnaire* (GQ-6; McCullough et al., 2002) in its German adaptation (Samson et al., 2011), using a seven-point Likert scale (1 = *strongly disagree*, 7 = *strongly agree*). It showed acceptable reliabilities in earlier studies (McCullough et al., 2002: α = 0.82; Samson et al., 2011: α = 0.73).

To record individuals’ general tendency for social comparison, we used the 11-item *Iowa-Netherlands Comparison Orientation Measure* (INCOM; Gibbons and Buunk, 1999) in its German version by Schneider and Schupp (2011). Social comparison tendency was assessed on a five-point Likert Scale (1 = *I disagree strongly*, 5 = *I agree strongly*), with a reliability (Cronbach’s α) ranging from 0.78 to 0.85.

For registering social media specific comparison processes we added the six-item *Social Comparison Tendency on Facebook Scale* (COM-F; Steers et al., 2014), translated into German and modified for Instagram by Meier and Schäfer (2018). It is referenced here as *COM-I* (Cronbach’s α = 0.85; Steers et al., 2014), using a five-point Likert scale as well.

Additionally, participants completed the *Benign and Malicious Envy Scale* (BeMaS; Lange and Crusius, 2015) for benign (α = 0.84–0.90) and malicious (α = 0.84–0.91) envy subtypes and general envy by calculating the total score. The envy scales were scored using a five-point Likert Scale (1 = *I disagree strongly*, 5 = *I agree strongly*). To gather Instagram specific envy, we applied the respective German adaptation by Meier and Schäfer (2018).

For registering inspiration, we used the *Inspiration Scale* (Thrash and Elliot, 2003) in its German adaptation (Meier and Schäfer, 2018) on a five-point Likert scale, showing Cronbach’s α’s between 0.90 and 0.95 (Thrash and Elliot, 2003).

As our final psychometric measure, we applied the widely used *Positive and Negative Affect Schedule* (PANAS; Watson et al., 1988; German version by Breyer and Bluemke, 2016) with α = 0.86 for both subscales. Again, a five-point Likert scale was used (1 = *very slightly or not at all*, 5 = *extremely*) to assess positive and negative affect with ten items each.

### 2.3. Procedure

Mainly, participants were recruited via social media, where an invitation and the link to the online-study were spread. No compensation for participation was offered. First of all, participants were asked to give informed consent for participation. Only then could they continue with filling out the respective questionnaires. The measures were presented in a fixed order to prevent differences in mutual interference of the measures for each participant. Contact details were presented in case any questions arose.

### 2.4. Data analysis

**FIGURE 1:**
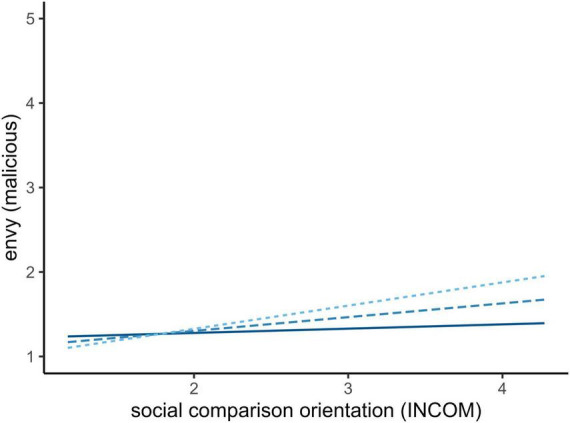
Simple slope analysis of gratitude on general social comparison. Full line (–1 SD) and fine-dotted line (+1 SD).

We excluded all participants with incomplete data or overly fast completion time (4 min). From 303 initially sampled individuals, 268 remained in the final sample. We initially calculated descriptive statistics and Cronbach’s α reliability coefficients for all scales and subscales used. To deliver a general oversight on the relationship between measurements, we obtained all correlations (see text footnote 1). To conduct the planned analysis, we tested the predictability of social orientation on envy and its subscales (benign and malicious) as well as the moderating role of gratitude on the relationship. This was done, using the general social orientation measure (INCOM) as well as the social media specified measure (COM-I). This procedure resulted in six models, which are described in Tables 4, 5. Wherever significant, the moderation is plotted, using simple slope analysis (Figures 1 (3). Last, we tested the consequences of benign and malicious envy on inspiration as well as positive and negative affect. Note that the term prediction is used non-causal in this article as the data does not allow for immediate causal conclusions. Rather, the term is used to describe explained variance. All reported regression coefficients are z-standardized.

### 2.5. Open science statement

The current study was not pre-registered, yet we follow the open science recommendations of open data and open code, which can be obtained from see text footnote 1.

## 3. Results

### 3.1. Descriptive statistics, reliabilities, and social media usage

Table 2 summarizes the descriptive statistics of the applied scales as well as their coefficient alphas. Apart from the GQ-6, all scales showed satisfactory reliability. The alpha of the scale states a limitation to the presented analysis.

**TABLE 2:** Descriptive details on applied scales.

	*M*	SD	Median	α
GQ-6	5.92	0.73	6.00	0.66 [0.61, 0.72]
INCOM	3.19	0.48	3.27	0.82 [0.78, 0.85]
COM-I	2.23	0.80	2.33	0.78 [0.74, 0.82]
Envy (general)	1.85	0.64	1.80	0.84 [0.81, 0.87]
Envy (benign)	2.21	0.94	2.20	0.87 [0.85, 0.89]
Envy (malicious)	1.49	0.62	1.20	0.79 [0.75, 0.83]
Inspiration	3.04	1.04	3.25	0.92 [0.91, 0.94]
Positive affect	3.19	0.63	3.30	0.83 [0.79, 0.86]
Negative affect	1.97	0.59	1.90	0.81 [0.77, 0.84]

Analysis of broad items regarding social media usage showed that 47.01% indicated to use it to gather information, 62.68% to interact with others, 88.06% for entertainment, and 49.63% for inspiration or motivation. Further items included distraction (54.47%), self-presentation (19.40%), keeping up-to-date (41.41%) as well as being creative (22.38%). The wording of items on additional information of Instagram usage style was chosen following Meier and Schäfer (2018).

Participants have been asked how much time they spend on Instagram per day (subjective estimation) which was answered by 265 participants. A total of 10.18% indicated 10 min or less, 24.53% reported 11–30 min, 33.96% said 31–60 min, 21.50% 1–2 h, 9.81% 2–3 h, and 0.02% more than 3 h per day.

As summarized in Table 3, participants were most likely to post self-related content (people and travel). The category *people* included selfies, group pictures, and portraits.

**TABLE 3:** Posted content categories by frequency in percentage (%).

	Almost never				Almost always
Fitness	77.61	11.19	7.08	2.61	1.49
Food	59.70	17.91	13.80	7.46	1.11
Fashion	86.94	5.97	4.85	1.49	0.74
People	18.65	14.92	16.41	25.74	24.25
Travel	17.91	8.20	20.52	33.95	19.40
Celebrities	91.41	7.08	1.11	0.37	0.00

### 3.2. Part I: predictability of envy and its facets

Following the planned analysis, overall envy as well as its subscales was predicted by the general tendency for social comparison. This prediction was significant and positive in all three models, indicating that social comparison tendencies relate toward higher experiences of envy (Table 4). For general and malicious envy, gratitude is negatively related to envy experiences, indicating a reversed relationship against social comparison tendencies. For malicious envy, social comparison tendencies and gratitude interacted on their effect on envy experience.

**TABLE 4:** Prediction of envy by general social comparison tendencies (INCOM).

	Envy					
	General		Benign		Malicious	
	β	CI	β	CI	β	CI
Intercept	0.01	−0.11, 0.12	0.01	−0.11, 0.12	0.01	−0.11, 0.13
INCOM	**0.26^∧^**	**0.14, 0.38**	**0.28^∧^**	**0.16, 0.40**	0.13*	**0.00, 0.25**
GQ-6	**−0.18^+^**	**−0.30, −0.06**	−0.08	−0.20, 0.04	−**0.25^∧^**	**−0.37, −0.13**
Interaction	−0.07	−0.16, 0.01	−0.05	−0.13, 0.04	−0.09*	**−0.17, −0.00**
*R*^2^ (adjusted)	0.100		0.081		0.072	

*p < 0.05, ^+^*p* < 0.01, ^∧^*p* < 0.001. Bold values indicates significance.

The simple slope analysis in Figure 1 indicates that individuals with higher mean gratitude (full-line) experience less malicious envy than individuals with less gratitude (fine-dotted line).

**FIGURE 2:**
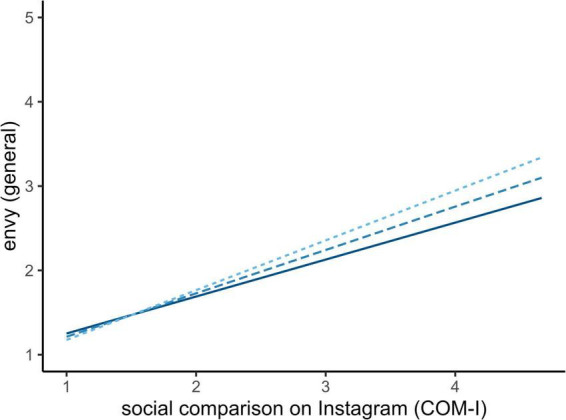
Simple slope analysis of gratitude on social media specific social comparison and general envy. Full line (–1SD) and fine-dotted line (+1SD).

**FIGURE 3:**
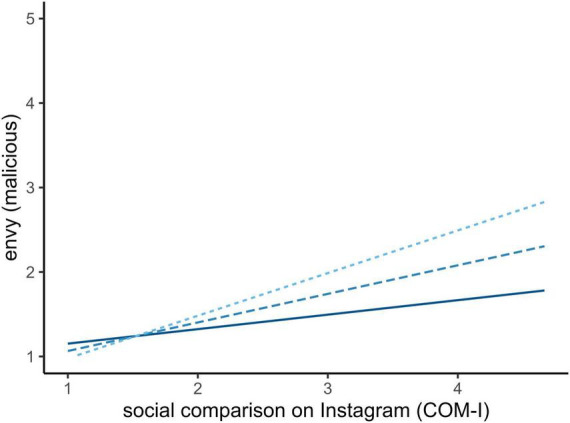
Simple slope analysis of gratitude on social media specific social comparison and malicious envy. Full line (–1SD) and fine-dotted line (+1SD).

When using the social media specific measure of social comparison (COM-I), we observed a very similar effect in directionality but higher explained variance across the models (Table 5). Again, social comparison predicted all facets of envy positively, yet meaningfully stronger. Gratitude moderated the relationship for general as well as malicious envy in the same directionality as before, where individuals with higher trait gratitude experience less envy (Figures 2, 3). It is noteworthy that there was no observed direct effect of gratitude but solely through the moderation of the effect of social comparison tendencies on envy.

**TABLE 5:** Prediction of envy by social media specific social comparison processes (COM-I).

	Envy					
	General		Benign		Malicious	
	β	CI	β	CI	β	CI
Intercept	−0.01	−0.10, 0.08	0.00	−0.10, 0.10	−0.01	−0.11, 0.08
COM-I	**0.64^∧^**	**0.55, 0.73**	**0.59^∧^**	**0.49, 0.69**	**0.44^∧^**	**0.34, 0.54**
GQ-6	−0.09	−0.18, 0.00	0.01	−0.09, 0.11	**−0.19^∧^**	**−0.29, −0.09**
Interaction	−0.09*	**−0.18, −0.01**	0.01	−0.08, 0.10	**−0.22^∧^**	**−0.31, −0.13**
*R*^2^ (adjusted)	0.448		0.337		0.316	

*p < 0.05, ^+^*p* < 0.01, ^∧^*p* < 0.001. Bold values indicates significance.

### 3.3. Part II: consequences of envy facets on inspiration and affect

To test the consequences of envy experience, we investigated the predictive value of benign and malicious envy on scales for inspiration as well as positive and negative affect (Table 6). The estimates underline the divergence of the envy dimensions. While inspiration and positive affect are negatively predicted by malicious envy, it is positively related to negative affect, indicating individuals who experience malicious envy tend to also experience less inspiration, less positive affect and more negative affect. On the other side, benign envy positively predicts inspiration and positive affect and was neutral to negative affect.

**TABLE 6:** Prediction of inspiration and affect by envy facets.

	Inspiration		Positive affect		Negative affect	
	β	CI	β	CI	β	CI
Intercept	0.00	−0.11, 0.11	−0.00	−0.12, 0.12	−0.00	−0.11, 0.11
Benign	**0.38^∧^**	**0.26, 0.50**	0.14*	**0.01, 0.26**	−0.01	−0.13, 0.11
Malicious	−0.16*	**−0.28, −0.04**	**−0.17^+^**	−**0.30,**−**0.05**	**0.33^∧^**	**0.21, 0.45**
*R*^2^ (adjusted)	0.122		0.025		0.098	

*p < 0.05, ^+^*p* < 0.01, ^∧^*p* < 0.001. Bold values indicates significance.

## 4. Discussion

### 4.1. Summary

In the present study, we investigated the relationship between social comparison tendencies and envy as well as the moderating role of dispositional gratitude. We observed that comparison tendencies and envy are related, on the overall level as well as by envy-facets (malicious and benign). Dispositional gratitude moderated this relationship, depending on the perspective on social comparison tendencies and envy conceptualization. Following, we explored the consequences of benign and malicious envy on subjective experiences of inspiration, positive and negative affect. This illustrates that malicious envy specifically holds potential for negative consequences.

### 4.2. Dispositional gratitude as a resource for social media use

When faced with the destructive aspects of Instagram use such as social comparison and envy, our findings highlight the potential of dispositional gratitude to serve as a buffer variable in this context. According to our results, dispositional gratitude significantly alters the relationship between social comparison and general/malicious envy on Instagram. Thus, gratitude can be considered a desirable trait for social media users. Therefore, the importance of promoting dispositional gratitude by interventions is no longer limited to real-life interactions, but transferrable to online-contacts too. Our work paves the way for further studies in this field, as it is crucial to shed light on the circumstances and individual characteristics that determine whether social media use results in favorable or else harmful outcomes. For this purpose, the moderation model proposed can be extended to complementary research questions.

#### 4.2.1. Specificity of social media directed comparison behavior

To our surprise, we observed differing results between the measures for social comparison. The predictive power (see β-values) underlines the stronger effect of social media specific comparison. In addition, only the effect of COM-I on general envy and malicious envy was moderated by individual gratitude. This might illustrate the relevance of context specific measures. As the INCOM measures the general tendency to socially compare oneself in the sense of an underlying traitlike variable, the scale was found to be a subject to socially desirable response behavior (Schneider and Schupp, 2011). The instructions and wording of the COM-I on the other hand refer to more state like reactions to opportunities for social comparison on Instagram specifically. Therefore, our findings form an interesting starting point for further investigations on media-specific measures as well as on the coherence of scales targeting the trait- vs. state-dimension.

#### 4.2.2. Differing effects of envy expression and corresponding effects on other resources

Concerning the continuing debate on how to conceptualize envy, we decided for an interim solution by differentiating between benign and malicious envy subtypes, but also investigating envy as a whole. Still, in the second part of our analysis we aimed at contributing to the discussion by validating the differing envy subtypes via their affective consequences and inspiration on Instagram. Benign envy positively predicted positive affect as well as inspiration on Instagram, while malicious envy positively predicted negative affect and negatively predicted inspiration. In combination with the findings of our moderation models, these results indicate the relevance of the dual envy conception. As our results show, different forms of envy are associated with opposing outcomes in terms of affect and inspiration. Omitting the analysis of envy facets would have resulted in a loss of information. Hence, our study suggests that the dual specification of envy allows an in-depth perspective on the data and enables the derivation of accurate conclusions on the harmful or else beneficial potential of envy. On that basis, not all manifestations of envy are to be considered detrimental ultimately.

### 4.3. Limitations and prospect

To capture the diverse realities of social media users, future research should focus on broader recruiting modalities, extending our approach to a more inclusive sample in terms of gender, age, and education. Generally, self-assessing online-surveys are prone to social desirability responding bias (van de Mortel, 2008). A more experience-based assessment method using online gratitude interventions could potentially lead to less biased responses. Another limitation is the low reliability of the GQ-6 in its German translation. This calls for supplementary validation studies on the GQ-6 in German samples.

Moreover, it has yet to be clarified how envy should effectively be conceptualized and measured. Based on the missing consensus, we see our method of analyzing subscales and the entire scale separately as adequate. Yet, we see that the construction as a two-factor latent construct or other measurement approaches could be valid as well.

To allow for a generalization of our findings, including other social media platforms is of further interest. Future studies in this field should supplement existing research by comparing different social network sites. Furthermore, the statistical model of moderation is inherently correlational and bidirectional, but theoretically deemed causal. Hence, it would be highly desirable for future research to implement an experimental or longitudinal design, as the moderating effect of gratitude is so far exclusively rooted in thorough theoretical derivation of presumed causality (Baron and Kenny, 1986; Wu and Zumbo, 2008).

## 5. Conclusion

Dispositional gratitude is a key trait variable in the context of social media use and a worthwhile research extension to that field. The high variance explained by the proposed moderation model emphasizes the role of gratitude as a protective factor for Instagram users. Future research should compare the moderating effect of gratitude on social media relevant variables before and after conducting online gratitude interventions. Additionally, the inclusion of clinically relevant variables such as depression or media addiction could be of interest for the extension of our model. By mitigating the relationship of social comparison and envy on Instagram, dispositional gratitude is a valuable resource for social media users. The enhancement of trait-gratitude through social-media-based intervention studies is therefore highly encouraged.

## Data availability statement

The datasets presented in this study can be found in online repositories. The names of the repository/repositories and accession number(s) can be found in the article/supplementary material.

## Ethics statement

The studies involving humans were approved by the Departmental Review Board, University of Vienna. The studies were conducted in accordance with the local legislation and institutional requirements. The participants provided their written informed consent to participate in this study.

## Author contributions

SK conceptualized the study, chose the methodology, collected and analyzed the data, and wrote the original draft as well as the revisions. LR curated and analyzed the data, and wrote and reviewed the manuscript. A-RL supervised the project and reviewed and edited the manuscript. All authors contributed to the article, going through multiple feedback loops and approved the submitted version.

## Footnotes

1 https://osf.io/qm9g5?view_only=d7596b60e9f648438612474233a97f78..
